# An unexpected case report of epidermoid cyst at the oculomotor nerve: mimicking a common cyst on MRI

**DOI:** 10.3389/fendo.2023.1153263

**Published:** 2023-06-14

**Authors:** Yuhao He, Sunfu Zhang

**Affiliations:** Department of Neurosurgery, Chengdu Third People’s Hospital, Chengdu, Sichuan, China

**Keywords:** intracranial lesions, epidermoid and dermoid cyst, oculomotor nerve, MRI, cyst < pathology

## Abstract

Intracranial epidermoid cysts are benign lesions and are rarely seen in clinical practice. Owing to similarities in imaging findings to those of common cystic lesions, the preoperative diagnosis is rendered challenging. Here, we present a case report of an epidermoid cyst at the right oculomotor nerve, which was initially misdiagnosed as a common cyst. A 14-year-old female child was admitted to our department due to a previous magnetic resonance imaging scan of a cystic lesion on the right side of the saddle that was suspected to be an oculomotor nerve cyst. In our department, this patient underwent a complete surgical resection of the tumor, and the pathology results revealed an epidermoid cyst. This is the first study that reported an epidermoid cyst at the right oculomotor nerve entering the orbit, mimicking a common cyst in imaging. We hope that this study would allow clinicians to consider this type of lesion as a differential diagnosis. Moreover, we suggest that specific diffusion-weighted imaging scan should be performed to aid in the diagnosis.

## Introduction

Intracranial epidermoid cysts are exceedingly rare and account for approximately 0.2%–1.8% of all intracranial tumors (1). Epidermoid cysts are congenital inclusion cysts derived from the remnants of ectodermal tissues misplaced during embryogenesis (2). Most of these reported lesions are located at the cerebellopontine angle (CPA), prepontine cistern, parasellar area, and middle cranial fossa (3) and can often cause neurological symptoms, such as hearing loss, vertigo, and trigeminal neuralgia (2). Surgery is generally performed for complete tumor removal, and the postoperative pathological diagnosis is regarded as the definitive diagnosis. Herein, we present a rare case of an epidermoid cyst located at the right oculomotor nerve, which was misdiagnosed due to the imaging findings and clinical history but was finally verified via histopathology.

## Case presentation

**Figure 1:**
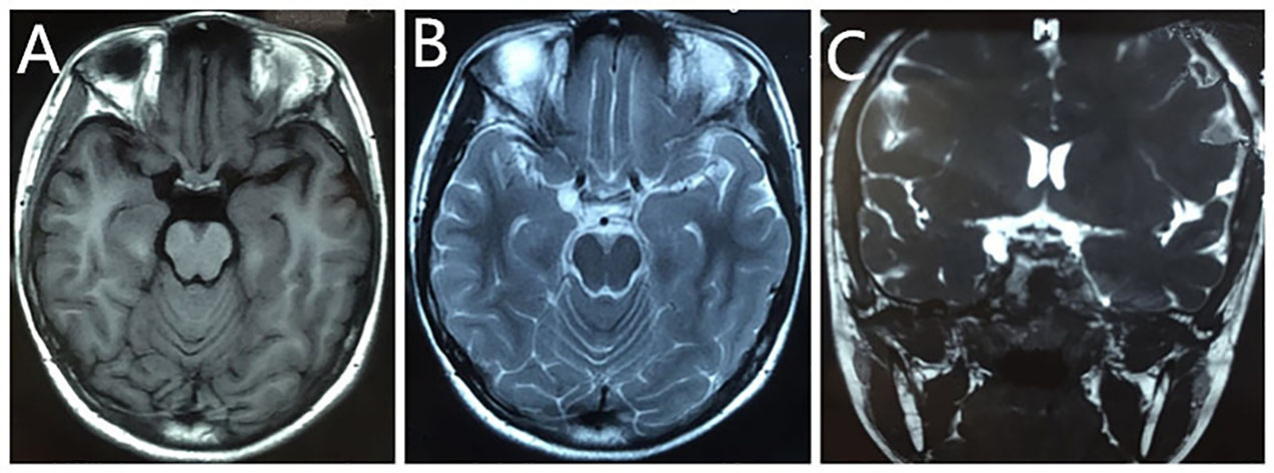
Preoperative MRI scan of the patient. T1-weighted images revealed a well-defined hypointense lesion at the right side of the saddle **(A)**. T2-weighted sequences showed a small cystic and homogeneous hyperintense lesion, measuring approximately 1.0 cm × 1.2 cm **(B)**. Cranial neuroimaging revealed a dumbbell-shaped cystic shadow on the right side of the saddle, suggesting a potential small cyst of the right oculomotor nerve root sheath **(C)**.

A 14-year-old female child was admitted to our hospital with complaints of sudden onset of dizziness, headache, visual deficit in the right eye, nausea, and vomiting for nearly 5 months. The patient’s past medical history was unremarkable. She underwent head magnetic resonance imaging (MRI) scan at a local hospital, which revealed a well-defined hypointense lesion at the right side of the saddle on T1-weighted images and a small cystic and homogeneous hyperintense lesion on T2-weighted sequences, measuring approximately 1.0 cm × 1.2 cm (**Figures 1A, B**), with a potential mass effect on the right oculomotor nerve. This lesion was associated with an abnormal light reflex. She also underwent cranial neuroimaging (**Figure 1C**), which demonstrated a dumbbell-shaped cystic shadow on the right side of the saddle, suggesting a potential small cyst on the right oculomotor nerve root sheath. In addition, she underwent cranial computed tomography (CT) scanning, and the imaging showed no abnormalities.

Thereafter, she was referred to our department due to an equivocal imaging diagnosis. Physical examination showed that the diameter of the right pupil was approximately 5 mm, and the direct and indirect light reflex was unappreciated. However, the diameter of the left pupil was approximately 3 mm, which was within the normal range and was sensitive to light reflexes. The laboratory blood tests showed the following: neutrophil at 86.1% (40%–75%), white blood cell count at 12.8 × 10^^9^/L (3.5–9.5 × 10^^9^/L), lymphocyte at 9.7% (20%–50%), and eosinophil at 0.0% (0.4%–8.0%). Moreover, the plasma adrenocorticotropic hormone was decreased at 2.39 ng/L (5.0–78 ng/L), and the sex hormone test revealed a decreased testosterone level with <0.03 ng/ml (0.07–0.78 ng/ml). The liver function tests were within the normal range. Considering the imaging features and the clinical manifestation, a primary diagnosis of a small cyst in the right oculomotor nerve root sheath or arachnoid cyst was established.

**Figure 2:**
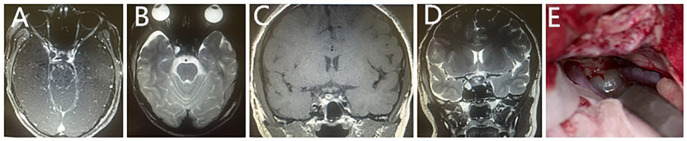
Postoperative MRI scan of the patient. T2-weighted imaging **(B, D)** showed an inhomogeneous signal on the right side of the pituitary gland, a nodular-like lesion that was hyperintense, measuring approximately 0.7 cm × 0.7 cm. On T1-weighted imaging **(C)**, it was hypointense, without enhancement after the intravenous administration of the extracellular contrast agent on postcontrast axial T1-weighted imaging **(A)**. **(E)** shows the gross specimen that was resected during the operation.

As the patient already underwent MRI and CT imaging at the local hospital, she therefore did not undergo another imaging examination at our hospital. Consequently, she was then scheduled for tumor resection on the right side of the saddle. During craniotomy, we found that the tumor was located on the right oculomotor nerve entering the orbit, which was characterized as cystic with a smooth surface and clear and transparent sac, approximately 1 cm × 1 cm in size (**Figure 2E**). Furthermore, a 0.5 cm × 0.5 cm white okara-like tissue at the right oculomotor nerve in the orbit, which is not adherent to the surrounding tissues and with an intact capsule, was observed.

The postoperative course was uneventful, and the post-MRI scan showed an inhomogeneous signal on the right side of the pituitary gland, a nodular-like lesion that was hyperintense on T2-weighted imaging (**Figures 2B, D**), measuring 0.7 cm × 0.7 cm, and was hypointense on T1-weighted imaging (**Figure 2C**), without enhancement after the intravenous administration of the extracellular contrast agent on postcontrast axial T1-weighted imaging (**Figure 2A**). The patient was discharged on the 12th postoperative day.

**Figure 3:**
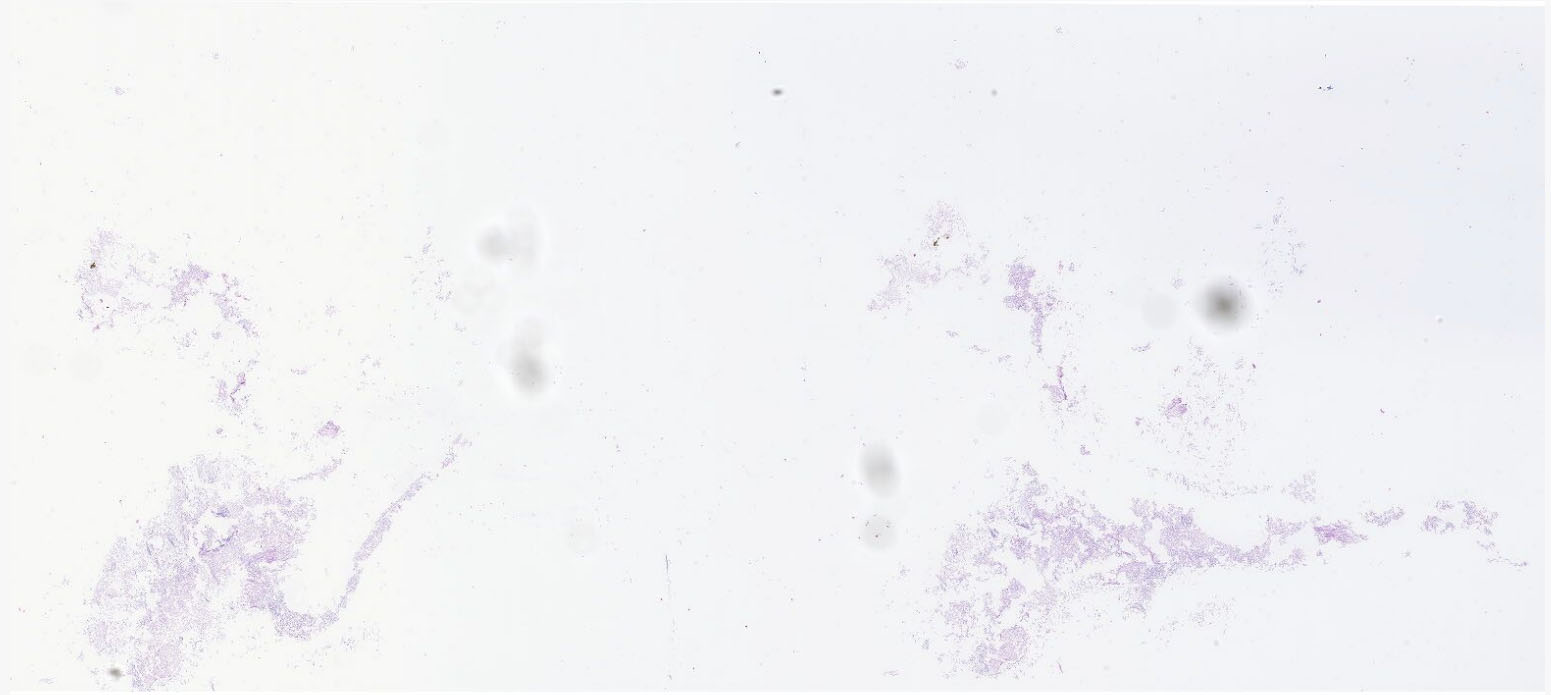
Specimens sent for pathological diagnosis revealed keratin debris without a squamous epithelial lining. Considering the intraoperative findings of this case, a definite diagnosis of an epidermoid cyst was established.

The pathology specimens revealed keratin debris without a squamous epithelial lining. Considering the intraoperative findings, a definite diagnosis of an epidermoid cyst was established (**Figure 3**).

## Discussion

We present a case of a child who complained of dizziness, headache, visual deficit in the right eye, nausea, and vomiting for nearly 5 months with an initial diagnosis of a common cyst at the right oculomotor nerve entering the orbit according to the imaging features and clinical manifestations. However, this lesion was finally confirmed to be an epidermoid cyst by pathological evaluation.

Most of the epidermoid cysts are benign extracerebral lesions due to the origin of the epithelium (4), and a few cases can undergo malignant transformation into a squamous cell carcinoma, of which the exact mechanism of this transformation remains unclear (5). No malignant transformation was observed after complete surgical resection, which was in line with most of the previous reports (3, 4, 6). In this case report, the patient presented with non-specific symptoms such as dizziness, headache, nausea, and disturbed light reflex of the right eye, which can be explained by the local mass effect of the tumor on adjacent brain parenchyma or cranial neurovascular structures (7). That is, the tumor encased the right oculomotor nerve resulting in the abnormal light reflex of this patient, which was in line with the observation that the tumor adhered firmly to the oculomotor nerve during surgery. The location of the tumor of this patient is a notable finding in this report. Indeed, previous studies have revealed that the most common location of this rare tumor is the CPA, followed by the prepontine cistern and parasellar area. A lesion located at the oculomotor nerve has not yet been reported, and a differential diagnosis of this unusual location occurring in the parasellar area should be provided.

The preoperative initial diagnosis of a common cyst was established according to imaging characteristics. Previous reports revealed varied MRI findings, possibly because of the different proportions of the components within the lesion (8). Specifically, on T1-weighted images, epidermoid cysts can appear as hypo-, iso-, or hyperintense, with or without rim enhancement following contrast administration. The iso- or hyperintensity on T1-weighted images can be explained by the extensive protein, lipid substances, cholesterol crystals in the capsule wall, and cyst contents (9, 10), and, on T2-weighted imaging, these lesions typically appear as hyperintense (4). However, most previous cases presented as hypointense on T1-weighted images and hyperintense on T2-weighted images. Similarly, in this case report, the patient presented with typical MRI features, which was congruent with the intraoperative findings. Moreover, we found a clear and transparent cystic fluid intraoperatively. This non-specific MRI manifestation also created a diagnostic dilemma; thus, a diagnosis of common cysts was initially established, followed by an arachnoid cyst, probably because epidermoid cysts are rarely seen in clinical practice.

As an important sequence, the diffusion-weighted imaging (DWI) of MRI can reflect the movement of water molecules in tissues and is often used to distinguish benign and malignant lesions in clinical practice. Notably, DWI can be effective in differentiating epidermoid cysts from arachnoid cysts, which shows the relative restriction of the diffusion of the epidermoid cysts as a characteristic feature, while arachnoid cysts show isointensity to cerebrospinal fluid on all magnetic resonance sequences without diffusion restriction (4, 11). However, in this patient, DWI scanning was not performed due to the misdiagnosis of a common cyst. Thus, DWI should be included as an imaging modality, which may be specific for epidermoid cysts when the preliminary diagnosis is equivocal.

Total resection of the epidermoid cysts by craniotomy is still the ultimate goal of management (12); however, in some cases, subtotal resection was performed because of the lesion’s firm adherence to the adjacent parenchyma or vital vasculature or nerve (13). The patient in this study underwent a complete resection of the lesion and had not complained of any postoperative deficits. However, in another case report of an epidermoid cyst located at the orbital apex, the patient had postoperative visual loss, which may be attributed to the manipulation of the optic nerve by the neurosurgeon to allow complete tumor resection (11). Thus, a comprehensive assessment of the lesion, as well as the surrounding structures, before surgery is required to achieve a better clinical outcome.

In conclusion, this is the first case report of epidermoid cysts located at the right oculomotor nerve entering the orbit and misdiagnosed as a common cyst. We hope that this case will aid clinicians in arriving at a clinical differential diagnosis; we also suggest that DWI scanning should be performed to help with the equivocal diagnosis. Due to its low occurrence and the lack of comprehensive assessment of this kind of lesion, more relevant evidence and case reports are needed to further elucidate this disease entity.

## Data availability statement

The raw data supporting the conclusions of this article will be made available by the authors, without undue reservation.

## Ethics statement

The studies involving human participants were reviewed and approved by Chengdu Third People’s Hospital. Written informed consent to participate in this study was provided by the participants’ legal guardian/next of kin. Written informed consent was obtained from the participant/patient(s) for the publication of this case report.

## Author contributions

YH contributed to the conception and design of the study, as well as the writing. SZ revised the manuscript. All authors contributed to the article and approved the submitted version.
